# Multiple endocrine neoplasia type 1 in childhood and description of a novel variant

**DOI:** 10.1590/1984-0462/2025/43/2024175

**Published:** 2025-03-24

**Authors:** Mayara Teixeira Alexandrino Sales, Rebeca Costa Castelo Branco, Carlos Henrique Paiva Granjeiro, Milena Silva Sousa, Luciana Felipe Férrer Aragão, Annelise Barreto de Carvalho, Ana Paula Dias Rangel Montenegro, Ana Rosa Pinto Quidute

**Affiliations:** a Universidade Federal do Ceará, Hospital Universitário Walter Cantídio, Fortaleza, CE, Brazil.

**Keywords:** Multiple endocrine neoplasia type 1, Insulinoma, Hypoglycemia, Child, Neoplasia endócrina múltipla tipo 1, Insulinoma, Hipoglicemia, Criança

## Abstract

**Objective:**

To describe a case of multiple endocrine neoplasia type 1 in the pediatric age group and its molecular diagnosis.

**Case description:**

An 11-year-old boy began to present generalized tonic-clonic seizures in the presence of hypoglycemia, with high insulin dosage, leading to suspicion of insulinoma. Abdominal magnetic resonance imaging confirmed a pancreatic nodule, which was surgically resected, resulting in glycemic normalization. Low growth hormone levels and hyperprolactinemia, secondary to macroprolactinoma, were also identified. Treatment with cabergoline led to a reduction in size. Hyperparathyroidism was found asymptomatically, with parathyroid scintigraphy suggestive of adenoma, thus, the patient underwent subtotal parathyroidectomy and thymectomy with resolution of the condition. He entered puberty spontaneously at 15 years of age; however, he had decreased growth speed, short stature, and low insulin-like growth factor 1 (IGF-1) levels, indicating recombinant growth hormone. The next-generation sequencing panel for multiple endocrine neoplasia type 1 identified a probably pathogenic variant c.442A>C: p.(Thr148Pro) in heterozygosity in the MEN1 gene, without previous description in databases (ClinVar).

**Comments:**

We highlight the pre-pubertal age of multiple endocrine neoplasia type 1 diagnosis, which is made before age 21 in only 12–17% of cases, and hypoglycemia secondary to insulinoma as the initial manifestation, differing from what is most frequently described, namely prolactinoma and parathyroid adenoma. The clinical diagnosis was made based on the occurrence of two primary endocrine tumors and confirmed through a next-generation sequencing panel, with a variant not previously described in ClinVar.

## RESUMO

**Objetivo:**

Descrever um caso de neoplasia endócrina múltipla tipo 1 na faixa etária pediátrica e seu diagnóstico molecular.

**Descrição do caso:**

Menino aos 11 anos passou a apresentar crises convulsivas em vigência de hipoglicemia, com insulina elevada, levando à suspeição de insulinoma. Ressonância magnética do abdômen confirmou nódulo pancreático, que foi ressecado cirurgicamente, resultando na normalização glicêmica. Foram identificados também baixos níveis de hormônio de crescimento e hiperprolactinemia, secundária à macroprolactinoma. O tratamento com cabergolina levou à redução do seu tamanho. De forma assintomática, foi constatado hiperparatireoidismo, com cintilografia das paratireoides sugestiva de adenoma, sendo o paciente submetido à paratireoidectomia subtotal e timectomia com resolução do quadro. Aos 15 anos, entrou em puberdade espontaneamente, no entanto, apresentava queda da velocidade de crescimento, baixa estatura e baixos níveis de fator de crescimento semelhante à insulina tipo 1 (IGF-1), com indicação de hormônio de crescimento recombinante. O painel de sequenciamento de nova geração para neoplasia endócrina múltipla tipo 1 identificou variante provavelmente patogênica c.442A>C: p.(Thr148Pro) em heterozigose no gene MEN1, sem descrição prévia em bancos de dados (ClinVar).

**Comentários:**

Destacamos a idade pré-puberal do diagnóstico da neoplasia endócrina múltipla tipo 1, que em apenas 12–17% dos casos é realizado antes dos 21 anos, e a hipoglicemia secundária ao insulinoma como manifestação inicial, diferindo do que é descrito mais frequentemente – prolactinoma e adenoma de paratireoides. O diagnóstico clínico foi feito com base na ocorrência de dois tumores endócrinos primários e confirmado por meio do painel de sequenciamento de nova geração, com variante sem descrição prévia no ClinVar.

## INTRODUCTION

Multiple endocrine neoplasia type 1 (MEN1) is a rare condition, prevalent in 3-20/100,000 people, characterized by the association of endocrine and non-endocrine tumors. It is a disease with an autosomal dominant inheritance pattern of high penetrance caused by pathogenic variants in the MEN1 tumor suppressor gene. The diagnosis is suspected due to the occurrence of more than one type of neoplasia in the parathyroid glands, anterior pituitary gland, and/or pancreas, in addition to possible association with thymic, pulmonary, gastric, and adrenocortical tumors.^1,2^

With increasing penetrance of the disease with age, clinical and biochemical manifestations are evident in 80% of patients and more than 98% in the fifth decade of life.^3^ A minority of cases (12–17%) of MEN1 are diagnosed in the first two decades of life, and even less frequently in adolescence.^1,4,5,6^ Thus, a case of MEN1 manifested at 11 years of age is reported herein due to the rarity of the disease and its onset in the pediatric age group.

## CASE REPORT

This case study was prepared based on the analysis of data gathered in the medical record after obtaining informed consent and assent, with approval by the Research Ethics Committee of the Hospital Universitário Walter Cantídio (CAEE 73962123.4.0000.5045).

**Figure 1:**
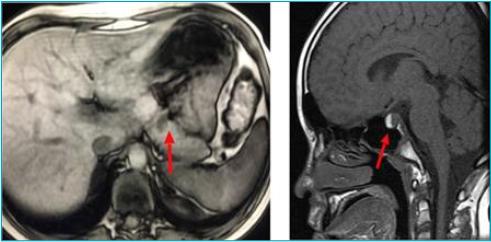
Magnetic resonance imaging of the A) abdomen: pancreatic nodule with hyposignal and well-defined limits (1.0 × 0.8 cm) in the T1out phase sequence without contrast – insulinoma; and B) sella turcica: lesion (10 × 12 mm) with hypersignal suggesting bleeding in the anterior compartment of the adenohypophysis in the T1 sequence without contrast – macroadenoma.

A boy with no prior pathological history, an adopted son, began to present generalized tonic-clonic seizures at the age of 11 and was recommended for treatment with anticonvulsants by the neurologist. However, tests to investigate the condition revealed a high insulin dosage in the presence of hypoglycemia (Table 1), and the patient was referred to a pediatric endocrinologist. At the time, he weighed 39.7 kg, had a height of 141.5 cm (standard deviation [SD] 1.73), a body mass index of 19.8 kg/m^2^ (SD +0.74), and reported several episodes of symptomatic hypoglycemia. Given the association of severe symptomatic hypoglycemia (tremors, sweating, and convulsions) and high insulin levels, the hypothesis of insulinoma was suggested and magnetic resonance imaging (MRI) of the abdomen was indicated, which identified a pancreatic nodule measuring 1.0 x 0.8 cm (Figure 1A), suggesting the diagnosis.

**Table 1:** Laboratory results of the blood sample collected during hypoglycemia (critical sample).

Fasting glucose (mg/dL)	Insulin (mU/L)	C-peptide (ng/mL)	ACTH (pg/mL)	Cortisol (mcg/dL)	IGF-1 (ng/mL)	GH (ng/mL)	Lactate (nmol/L)	Uric acid (mg/dL)	Ketonuria	AST/ALT (U/L)
33	12.1	2.99	63.8	25	125	1.24	2.1	3.1	Negative	17/7

ACTH: adrenocorticotropic hormone; IGF-1: insulin-like growth factor-1; GH: growth hormone; AST: aspartate aminotransferase; ALT: alanine aminotransferase.

Low growth hormone (GH) levels were also identified in the critical sample. MRI of the sella turcica showed a 1.0 x 1.2 cm pituitary macroadenoma with signs of bleeding inside, causing a shift of the pituitary stalk and pituitary gland to the left (Figure 1B). Hyperprolactinemia (348.4 ng/ml) was also found, with a recovery percentage of 90% after ethylene glycol. Considering the association of these two endocrine tumors, the patient received a clinical diagnosis of MEN1 and underwent surgical resection of the insulinoma and drug treatment of the prolactinoma with cabergoline at a dose of 0.75 mg/week. The child progressed with normalization of blood glucose levels and a reduction in the size of the pituitary adenoma and prolactin levels to 37.4 ng/ml (reference value [RV] 2.1–20.3 g/ml). The initial exams were complemented with a gastrin dosage of 22.5pg/ml (RV 13–115pg/ml) and chromogranin A of 108 ng/ml (RV<108 ng/ml), ruling out other enteropancreatic tumors.

**Figure 2:**
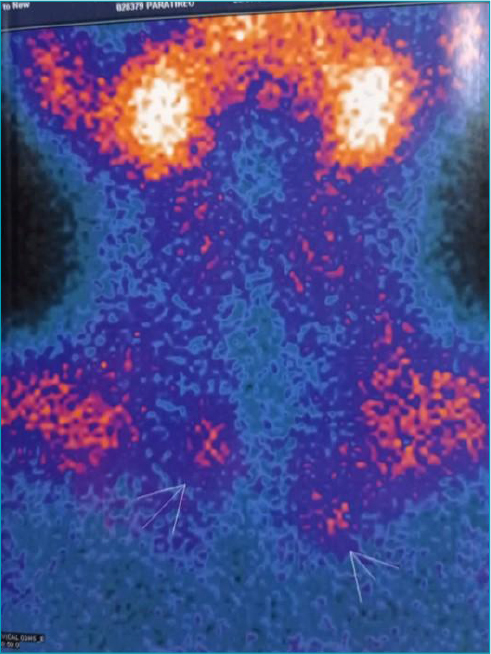
Thyroid and parathyroid scintigraphy with areas of late radiopharmaceutical retention in the topography of the lower thirds of the right and left lobes/cervicothoracic transition suggestive of adenoma of the lower parathyroid glands.

During clinical and laboratory monitoring at the age of 13, hypercalcemia (1.57 nmol/l), hypophosphatemia (3.7 mg/dl), calciuria 34.5 mg/dl (RV 6–21 mg/dl) were identified asymptomatically, and parathyroid hormone (PTH) dosed at 91.3pg/ml (RV 10–68 pg/ml). Ultrasonography of the kidneys and urinary tract showed bilateral nephrocalcinosis, and parathyroid scintigraphy revealed late retention areas of the radiopharmaceutical suggestive of adenoma (Figure 2). With these findings of primary hyperparathyroidism, he underwent subtotal parathyroidectomy (three parathyroid resections) and thymectomy, resulting in the normalization of serum calcium, phosphorus, and PTH levels. Then he entered puberty spontaneously at the age of 15; however, he had decreased growth speed, short stature (SD 2.6), and IGF-1 of 118ng/ml (RV 177–507 ng/ml), thus, treatment with recombinant GH was indicated.

The next-generation sequencing (NGS) panel for MEN1 identified the probably pathogenic variant c.442A>C: p. (Thr148Pro) in heterozygosity in the MEN1 gene. This variant is not currently described in databases (ClinVar).

## DISCUSSION

We highlight in the reported case the pre-pubertal age of MEN1 diagnosis, which is made before age 21 years in only 12–17% of cases, with even lower rates before adolescence. In this case, we followed the recommendation to research the mutation in individuals who present tumors associated with MEN1 at an early age. It is also suggested that genetic testing be carried out on asymptomatic first-degree relatives of patients with a mutation in the MEN1 gene as quickly as possible.^1^

The classic endocrine disorders of MEN1 may be the first manifestation of the syndrome in this age group, with functional tumors being diagnosed earlier.^1^ For some authors, prolactin-secreting tumors are the most common. However, primary hyperparathyroidism was the most prevalent manifestation (75%) among young patients in a cohort of 924 patients with MEN1, followed by pituitary adenomas (34%) and pancreatic neuroendocrine tumors (34%), with the majority of the latter not being functional.^2^ In this reported case, hypoglycemia, secondary to insulinoma, was the first clinical manifestation.

The diagnosis of MEN1 is made by the presence of at least one of the following criteria:

1. Occurrence of two or more primary endocrine tumors associated with MEN1 (e.g., parathyroid adenoma, enteropancreatic tumor, and/or pituitary adenoma);
2. Occurrence of one of the MEN1-associated tumors in a first-degree relative of an individual with a clinical diagnosis of MEN1; or
3. Identification of a MEN1 germline mutation in an individual who may be asymptomatic and has not yet developed biochemical or radiological serum abnormalities indicative of tumor development.^2^

The clinical diagnosis of this case was made based on the occurrence of two primary endocrine tumors (insulinoma and prolactinoma) and confirmed through an NGS panel, with the description of a novel variant.

According to the 2012 Clinical Practice Guidelines for Multiple Endocrine Neoplasia Type 1,^3^ biochemical screening for pituitary tumors for individuals at high risk of MEN1 (i.e., mutation carriers) should include assessment of plasma prolactin and IGF-1 levels annually, in addition to MRI or computed tomography (CT) of the pituitary gland every 1–3 years. In case of abnormal results, measurement of other hormones and dynamic tests must be performed. Screening for primary hyperparathyroidism should include annual plasma calcium and PTH measurements. Screening for enteropancreatic neuroendocrine tumors (NET) should also be conducted annually with the hormonal profile of the gastrointestinal tract, including measurement of glucose, insulin, glucagon, gastrin, and chromogranin A. MRI, CT, or endoscopic ultrasound are also recommended on an annual basis.

Treatment of pituitary tumors is similar to that of sporadic tumors; it consists of the use of drugs, such as dopaminergic agonists for prolactinoma and octreotide for somatotropinomas, or the procedure of selective transsphenoidal hypophysectomy, with radiotherapy reserved for unresectable residual tumor tissue. Surgery is the treatment of choice in the case of parathyroid hyperplasia, which may be subtotal (at least three glands) or total parathyroidectomy. Concomitant transcervical thymectomy is also recommended. The treatment of functioning and symptomatic NETs, including insulinoma, aims to achieve a cure through surgery.^3^

Despite advances in the diagnosis and treatment of MEN1, life expectancy is reduced compared to the rest of the population, with the average age of death of 55–60 years, with the cause in 50–70% of cases being related to the disease itself.^2^ Enteropancreatic tumors, such as gastrinomas and carcinoid tumors, are the main causes of death in patients with MEN 1.^5^

## Data Availability

The database that originated the article is available with the corresponding author.
